# Effects of Salinity on Physicochemical Properties, Flavor Compounds, and Bacterial Communities in Broad Bean Paste-Meju Fermentation

**DOI:** 10.3390/foods13132108

**Published:** 2024-07-02

**Authors:** Qingyan Guo, Jiabao Peng, Jingjing Zhao, Jie Lei, Yukun Huang, Bing Shao

**Affiliations:** 1 Food Microbiology Key Laboratory of Sichuan Province, School of Food and Bioengineering, Xihua University, Chengdu 610039, China; 19150356036@163.com (J.P.); zhaojingjingly@163.com (J.Z.); 0120210019@mail.xhu.edu.cn (J.L.); hyk_diana@163.com (Y.H.); shaobing@163.com (B.S.); 2 Chongqing Key Laboratory of Speciality Food Co-Built by Sichuan and Chongqing, Chengdu 610039, China; 3 Beijing Key Laboratory of Diagnostic and Traceability Technologies for Food Poisoning, Beijing Center for Disease Prevention and Control, Beijing 100013, China

**Keywords:** broad bean paste, salinity, microbial community, flavor compounds, correlation

## Abstract

Broad bean paste (BBP) is a traditional fermented soy food, and its high salt content not only prolongs the fermentation time but also threatens human health. In this study, three BBP-meju with different salt concentrations were prepared, and the effects of varying salinity on fermentation were comprehensively compared. The results showed that salt-reduced fermentation contributed to the accumulation of amino acid nitrogen, reducing sugars, free amino acids, and organic acids. Alcohols, esters, aldehydes, and acids were the main volatile flavor compounds in BBP-meju, and the highest total volatile flavor compounds were found in medium-salt meju. *Bacillus*, *Staphylococcus*, *Aspergillus*, and *Mortierella* were the dominant microbial communities during fermentation, and there were also three opportunistic pathogens, *Enterobacter*, *Pantoea*, and *Brevundimonas*, respectively. According to Spearman correlation analysis, *Wickerhamomyces*, *Bacillus*, *Staphylococcus*, and *Mortierella* all showed highly significant positive correlations with ≥3 key flavor compounds, which may be the core functional flora. Furthermore, the dominant microbial genera worked synergistically to promote the formation of high-quality flavor compounds and inhibit the production of off-flavors during salt-reduced fermentation. This study provides a theoretical reference for the quality and safety control of low-salt fermented soy foods.

## 1. Introduction

In recent years, there has been an increasing consumer demand for rational diets. Fermented soy foods have garnered widespread attention globally due to their nutritional and health benefits [1]. Broad bean paste (BBP), also known as Pixian-douban or Doubanjiang, is a primary traditional fermented soy food in China, recognized as a Chinese geographical indication protected product and a national intangible cultural heritage [2]. The production of BBP mainly involves three stages: (1) chopped chili peppers are naturally fermented to produce moromi, (2) broad beans are inoculated with *Aspergillus oryzae* and then converted to meju via fermentation, and (3) the moromi-meju mixture is exposed to sunlight, sheltered from rain, regularly stirred during the day and left to ferment through dew at night until mature [3]. During the BBP-meju fermentation, vigorous microbial metabolism converts macromolecules such as proteins and starch into amino acids, peptides, flavonoids, and free sugars, imparting rich flavor and various physiological functions, which significantly impact the final quality of BBP [4,5]. Reports suggested that BBP exhibited excellent anti-inflammatory, antioxidant, anti-hypertensive, and anti-obesity functions [6,7]. However, according to the Chinese national standard, the salinity of commercial BBP is 15–22% (*w*/*w*), while the amount of salt added in BBP-meju is 12–13% (*w*/*w*) [8]. Numerous studies indicated that a high-salt diet could induce hypertension, osteoporosis, cardiovascular and cerebrovascular diseases, and kidney diseases [9,10], and was also a risk factor for Alzheimer’s disease [11]. The World Health Organization (WHO) recommends a daily sodium intake of 2 g for adults, equivalent to 5 g of salt per day [12]. Additionally, the WHO released the “WHO global sodium benchmarks for different food categories (2nd edition)” [13] in 2024. It serves as a guide for countries and industries to reduce sodium content in various processed foods, aiming to collectively achieve the WHO’s goal of reducing global salt/sodium intake by 30% by 2025 [14]. Moreover, the “Chinese National Nutrition Plan (2017–2030)” explicitly states the goal of reducing salt intake among Chinese residents by 20% by 2030 [15]. Therefore, reducing BBP salt concentration is crucial to promote further development in this industry.

Currently, strategies used for salt reduction in BBP are mainly based on the replacement of NaCl by other substances, including CaCl_2_, MgCl_2_, KCl, yeast extracts, and phosphates [16]. Research indicated that increasing potassium intake through diet can alleviate symptoms in hypertensive patients induced by sodium [17]. This was primarily manifested by a reduction in urinary calcium excretion as well as a protective effect on skeletal mass [18]. However, fermented foods produced as substitutes using potassium or calcium salt contained bitter and unpleasant off-flavors that severely restricted their consumer acceptance [19,20]. Meanwhile, since osmotic pressure under high-salt conditions can inhibit the growth of spoilage microorganisms, most low-salt fermented foods require additional preservatives [21], affecting their safety and health. Therefore, salt reduction in fermented foods must be carried out with great caution. Studies have shown that salt-reduced fermentation accelerates BBP fermentation, promotes the production of organic acids (OAs) and free amino acids (FAAs), and increases the accumulation of flavor compounds and alkaloids [4]. Nevertheless, a decrease in salt concentration causes an increase in undesirable flavor compounds such as acetic acid, butyric acid, 2-phetylfuran, decanal, and (Z)-2-heptenal, which makes BBP exhibit acidity and irritation [22]. Furthermore, salt reduction during BBP fermentation can increase *Klebsiella*, *Cronobacter*, and *Acinetobacter* [23]. They are opportunistic pathogens that pose a food safety threat to BBP. Therefore, understanding the role of salinity on microorganisms and flavor compounds during BBP fermentation is crucial for achieving a salt reduction in BBP. Currently, BBP-related studies have focused on the flavor profile, microbial community structure, and functional properties [24], while the influence of salt concentration on BBP, especially during the fermentation process, remains unclear.

Based on the critical role of the BBP-meju fermentation stage, three BBP-meju with different salt concentrations were prepared in this study. The impact of salt concentration on BBP-meju quality was comprehensively explored through E-nose, headspace solid-phase microextraction combined with comprehensive two-dimensional gas chromatography-mass spectrometry (HS-SPME-GC × GC-MS) technology, and high-throughput sequencing technology, integrating physicochemical indexes, volatile flavor compounds, and microbial community succession. Furthermore, the relationship between key microbial communities and flavor compounds during salt-reduced fermentation was elucidated through correlation network analysis, providing theoretical guidance for the industrial production of low-salt BBP. Meanwhile, the results can guide the salt reduction strategies for traditional fermented soy foods similar to BBP, contributing to the reduction in global salt intake.

## 2. Materials and Methods

### 2.1. Fermentation of Broad Bean Paste-Meju under Different Salt Concentrations

Freshly shelled broad beans were soaked and boiled in water at high temperatures (115 °C) and high pressure (0.1 MPa) for 20 min using an electric pressure cooker (SY-30YZ100, SUPOR, Hangzhou, China). The cooked broad beans were fished, drained, and cooled to room temperature. Then, they were mixed with wheat flour sieved through 80 mesh at 3:1 (*w*/*w*), and then added with *Aspergillus oryzae* HN3.042 spore suspension to give a final inoculum of 1 × 10^7^ CFU/g. The mixture was incubated at 35 °C, 90% humidity for 48 h until green spores coated the surface beans, resulting in mature koji. Different salt concentrations were added to the matured koji to give a final concentration of 4%, 8%, and 12% (*w*/*w*) (defined as low, medium, and high concentrations, respectively). This mixture was transferred to a ceramic jar for anaerobic fermentation, sealed with distilled water, and left at 30 °C for 49 days. The samples were collected on days 0, 3, 5, 7, 14, 21, 28, 35, 42, and 49.

In total, 5 g of the BBP-meju sample was accurately weighed, and the distilled water was added to a total volume of 50 mL, homogenized, and then extracted with ultrasound at 35 °C for 30 min. Centrifuged at 10,000× *g* for 15 min at 4 °C, the supernatant was filtered through a 0.45 μm microporous filter, and the filtrate was collected. The filtrate was freeze-dried and diluted with distilled water for subsequent analysis.

### 2.2. Physicochemical Properties

Direct desiccation was employed to determine the moisture content. The pH was assessed utilizing a pH meter (METTLER TOLEDO Instruments Co., Shanghai, China). Salinity was determined via the silver nitrate titration [25]. Total acidity was titrated with sodium hydroxide [26]. The amino acid nitrogen (AAN) content analysis was carried out using the formaldehyde titration method [27]. Reducing sugars were quantified using the 3,5-dinitrosalicylic acid technique [28], while total sugars were determined with the phenol-H_2_SO_4_ technique [29].

The FAA content of BBP-meju samples was analyzed following the method outlined by Ding et al. with slight modifications [30]. The samples (5 g) were homogenized thrice for 10 s in 20 mL of distilled water at 10,000 g. Subsequently, 20 mL of 5% (*v*/*v*) trichloroacetic acid was added and mixed thoroughly. The mixture was then filtered through a 0.22 μm filter, and the FAA concentration was determined using an automatic amino acid analyzer (L-8900, Hitachi, Tokyo, Japan).

OAs were measured using HPLC (LC-16, Shimadzu, Kyoto, Japan) with Venusil MPc18 (250 mm × 4.6 mm, 5 μm) (Agela Technologies Co., Tianjin, China) and an SPD-16 detector (Shimadzu, Japan) as per the method developed by Lin et al. [31].

### 2.3. E-Nose Analysis

An E-nose analysis was conducted using a portable electronic nose 3 (PEN3) system (Ensoul Technology Ltd., Beijing, China) equipped with 10 MOS gas sensors. The detailed information of each sensor is shown in Table 1. Two g of BBP-meju samples were accurately weighed and placed in the headspace vials and equilibrated in a constant temperature water bath at 25 °C for 30 min. The parameters of the E-nose were set as follows: sampling interval of 1 s, cleaning time of 120 s, zero-point adjustment time of 10 s, pre-sampling time of 5 s, testing time of 80 s, and an injection volume flow rate of 400 mL/min. Each test group was measured in triplicate, and the average value was taken for data analysis [32].

### 2.4. Volatile Flavor Compounds Analysis

#### 2.4.1. SPME Method

In total, 2 g of BBP-meju sample was mixed with 2 g of NaCl and added to 8 mL of distilled water, followed by 50 μL of 2-octanol as an internal standard (0.822 mg/mL dissolved in distilled water) [33,34]. The mixture was placed into a headspace vial (20 mL) (Supelco, Inc., Bellefonte, PA, USA). Then, the headspace vial was ultrasonicated in a water bath at 30 °C for 30 min, followed by ramping up to 60 °C and equilibrium for 25 min, inserting a 75 μm SPME fiber (50/30 μm DVB/CAR/PDMS) (Supelco, Inc., Bellefonte, PA, USA) for extraction for 25 min. The fiber was then desorbed for 1 min and inserted into the GC injection port.

#### 2.4.2. GC × GC-MS Instrumental Analysis Method

The analysis was performed using a comprehensive two-dimensional gas chromatography-mass spectrometer (GCMS-QP2020NX, Shimadzu, Kyoto, Japan).

The injection port temperature was 230 °C, and the injection was carried out in non-split mode using helium (purity > 99.9999%) as the carrier gas with a column flow rate of 1 mL/min. The 1D column was a DB-WAX quartz capillary column (30 m × 0.25 mm × 0.25 μm), and the 2D column was a DB-17MS capillary column (1.2 m × 0.18 mm × 0.18 μm), along with a solid-state thermal modulator HV (C5–C30). The temperature program was as follows: started at 40 °C and held for 3 min, ramped up to 150 °C at a rate of 4 °C/min and held for 2 min, then ramped up to 230 °C at a rate of 4 °C/min and held for 6 min. The modulation cycle for the two-dimensional analysis was 4 s. The source temperature of the mass spectrometer was 230 °C, the interface temperature was 200 °C, the data acquisition was performed in SCAN mode for qualitative analysis, and the scanning range was 41–330 *m*/*z*.

The volatile compounds were identified by comparing retention index (RI) and mass spectra with reference standards from the National Institute of Standards and Technology (NIST) mass spectral library. Subsequently, quantitative analysis was carried out using 2-octanol as an internal standard. The relative concentrations of volatile compounds were then calculated by comparing the peak areas with the internal standard.

### 2.5. DNA Extraction and High-Throughput Sequencing Analysis

Microbial DNA was extracted following the protocol provided by the E.Z.N.A^®^ soil DNA kit (OmegaBio-Tek, Norcross, GA, USA). Subsequently, DNA quality, concentration, and integrity were evaluated using 1% agarose gel electrophoresis. The amplification of the V3-V4 region of the bacterial 16S rRNA gene employed primers 338F (5′-ACTCCTACGGGGAGGCAGCA-3′) and 806R (5′-GGACTACHVGGGTWTCTAAT-3′), while amplification of the fungal ITS gene sequences utilized primers ITS1F (5′-CTTGGTCATTTAGAGAGGAAGTAA-3′) and ITS2R (5′-GCTGCGTTCTTCATCGATGC-3′). PCR amplification followed the method outlined by Xu et al. [35]. Following purification, quantification, and storage at −80 °C, amplification products underwent raw sequencing data filtering by Trimmomatic v0.33 software. Primer sequences were eliminated using cutadapt 1.9.1 software to obtain high-quality sequences (Clean Reads). Subsequently, sample clean reads were spliced using Usearch v10 software and length-filtered according to specific region length ranges. The data underwent the removal of low-quality and chimeric sequences and the classification of high-quality sequences. The OTU representative sequences were taxonomically classified utilizing the Silva 138 16S rRNA database (http://www.arb-silva.de, URL (accessed on 23 April 2024)) and the Unite 8.0 ITS database (http://unite.ut.ee/, URL (accessed on 23 April 2024)) for taxonomy annotation.

### 2.6. Statistical Analysis

Three identical experiments (*n* = 3) were established for each trial. Statistical analysis was conducted through one-way ANOVA and Duncan’s multiple range test utilizing SPSS 25.0 software (SPSS Inc., Chicago, IL, USA). A significance level of 0.05 was employed to determine statistically significant variances. The research findings were represented graphically using Origin 2024 software (OriginLab Corporation, Northampton, MA, USA).

## 3. Results and Discussion

### 3.1. Physicochemical Properties during Fermentation of BBP-Meju with Different Salt Concentrations

**Figure 1:**
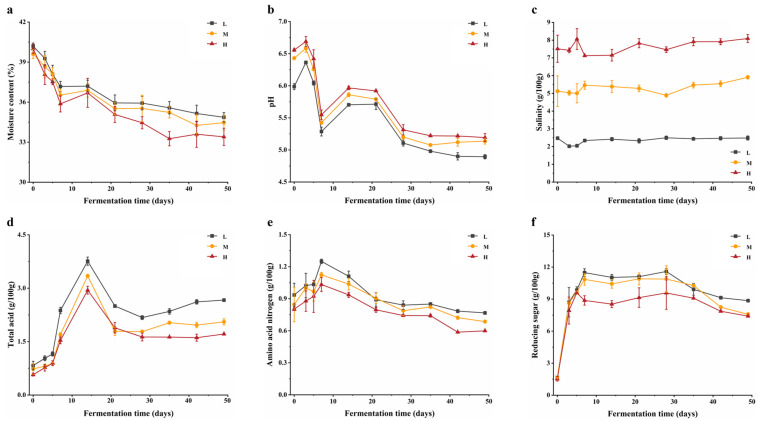
Physicochemical properties during fermentation of BBP-meju with different salt concentrations: (**a**) moisture contents; (**b**) pH; (**c**) salinity; (**d**) total acid contents; (**e**) AAN contents; (**f**) reducing sugar contents.

Physicochemical parameters are vital indicators affecting the quality of fermented soy foods. In this study, moisture content, pH, salinity, total acid, AAN, and reducing sugar during the fermentation of BBP-meju with different salt concentrations were determined (Figure 1). The salinity of the 4%, 8%, and 12% salt addition samples was stable and reached 2.48 ± 0.12 g/100 g, 5.91 ± 0.10 g/100 g, and 8.10 ± 0.22 g/100 g, respectively, on day 49 (Figure 1c). The moisture content of the BBP-meju with different salt concentrations decreased during fermentation, with higher moisture content observed with lower salt concentrations (Figure 1a), consistent with Li et al. [36]. The research indicated that the moisture content is a crucial indicator during the fermentation of BBP, which provides a transport medium for microbial growth and participates in microbial metabolism and reproduction. Therefore, the moisture content affects microbial activity and the occurrence of Maillard reactions in the fermentation system [37]. The higher moisture content in low-salt samples reflected more vigorous microbial growth and metabolism internally. Additionally, the pH of BBP-meju decreased first, then increased, and decreased again, stabilizing at 4.89 ± 0.03 (L), 5.14 ± 0.04 (M), and 5.19 ± 0.06 (H), respectively (Figure 1b), showing an opposite trend to the total acid. Overall, pH increased as salinity decreased, while total acid levels decreased. The total acidity levels of low- and medium-salt samples reached 2.67 ± 0.01 g/100 g and 2.06 ± 0.09 g/100 g, respectively (Figure 1d). They both exceeded the requirement of total acid (in terms of lactic acid) ≤ 2.0 g/100 g as specified in the Chinese national standard [8]. It is known that salt-reduced fermentation promotes the growth of microorganisms such as LAB, *Staphylococci*, and *Bacillus*. Further breakdown of carbohydrates, proteins, and fats in the raw materials leads to the accumulation of OAs. This ultimately results in an increase in total acid levels and a decrease in pH [30]. Meanwhile, pH is considered a critical factor in controlling the growth of pathogens in fermented foods and a vital hygiene indicator for BBP [38]. Therefore, lower salt concentrations can be used to some extent to achieve inhibition of pathogenic microorganisms through low pH. AAN is another important parameter for measuring the quality of BBP, reflecting the degree of hydrolysis of broad bean protein during fermentation [39]. The results showed that in the early stage of fermentation, the AAN levels in BBP-meju samples showed an increasing trend and reached the highest value on day 7, which was 1.25 ± 0.02 g/100 g (L), 1.12 ± 0.02 g/100 g (M), and 1.03 ± 0.07 g/100 g (H), respectively (Figure 1e). In the middle and late stages of fermentation, the AAN levels decreased gradually and reached 0.77 ± 0.01 g/100 g (L), 0.69 ± 0.01 g/100 g (M), and 0.60 ± 0.01 g/100 g (H) on day 49 (Figure 1e). In the early stage of fermentation, broad bean protein underwent extensive hydrolysis by microorganisms, leading to a rapid increase in AAN. However, in the middle and late stages of fermentation, the growth of microorganisms and the Maillard reactions consumed a large amount of AAN. This resulted in a decrease in the AAN content in BBP-me, which in turn led to a change in the flavor of BBP [40]. Similarly, microorganisms such as *Aspergillus oryzae* secreted large amounts of amylase to degrade starch in broad beans, causing a rapid increase in the level of reducing sugars in the early stages of fermentation. In the middle and late stages of fermentation, reducing sugars were consumed as a substrate for microbial growth and the Maillard reaction, leading to a decrease in reducing sugars [41]. Ultimately, on day 49, it reached 8.86 ± 0.01 g/100 g (L), 7.57 ± 0.17 g/100 g (M), and 7.41 ± 0.03 g/100 g (H), respectively (Figure 1f). Overall, the salt reduction caused the accumulation of AAN and reducing sugars, positively affecting the overall quality of BBP-meju. In addition, multiple physicochemical indicators used day 7 as a critical turning point, followed by continuous fluctuations. Therefore, the fermentation samples from days 7, 21, 35, and 49 were selected for subsequent analysis.

### 3.2. Changes in FAA Composition during Fermentation of BBP-Meju with Different Salt Concentrations

**Figure 2:**
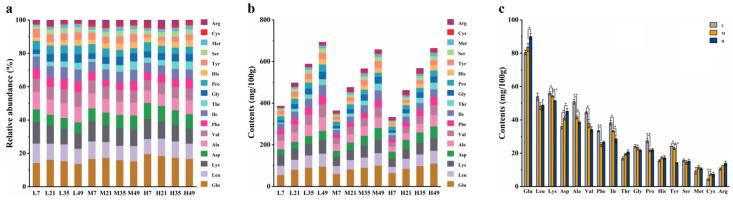
FAAs during fermentation of BBP-meju with different salt concentrations: (**a**) content of amino acids; (**b**) relative abundance of amino acids; (**c**) average content of amino acids in BBP-meju samples. Asterisks indicate a statistically significant difference from the control group (* *p* ≤ 0.05, ** *p* ≤ 0.01).

FAAs are crucial flavor compounds in fermented foods, closely associated with BBP-meju fermentation [42]. The changes in FAAs during BBP-meju fermentation with different salt concentrations are shown in Figure 2. It can be seen that the total amount of FAAs increased with fermentation at all three salt concentrations (Figure 2b). Meanwhile, the total amount of FAAs decreased slightly with increasing salt concentration, indicating that salt-reduced fermentation could further promote the accumulation of amino acids in BBP-meju. Studies have shown that salt reduction can enhance protease activity, resulting in higher levels of FAAs [43]. This is in agreement with the results of this study. Specifically, the levels of umami amino acids (glutamate and aspartate) decreased with salt reduction, with a total reduction of 16.04% (Figure 2a). On average, the glutamate (Glu) level decreased from 90.00 ± 1.88 mg/100 g in the high-salt sample to 80.43 ± 1.44 mg/100 g in the low-salt sample, while the aspartate (Asp) level decreased from 45.20 ± 1.71 mg/100 g in the high-salt sample to 36.09 ± 1.57 mg/100 g in the low-salt sample (Figure 2b). Similarly, the sweet amino acid threonine (Thr) level decreased with salt reduction. However, the levels of alanine (Ala), glycine (Gly), and proline (Pro) increased with salt reduction by 31.76%, 11.25%, and 24.35%, respectively (Figure 2c). Consequently, the total amount of sweet amino acids increased by 14.06% with salt reduction (Figure 2a). Likewise, the total amount of bitter amino acids showed an increasing trend, with an increase of 16.30% (Figure 2a). Thus, the salt reduction during BBP-meju fermentation led to a decrease in umami amino acids and an increase in bitter amino acids, resulting in flavor dissonance.

### 3.3. Changes in OA Composition during Fermentation of BBP-Meju with Different Salt Concentrations

**Figure 3:**
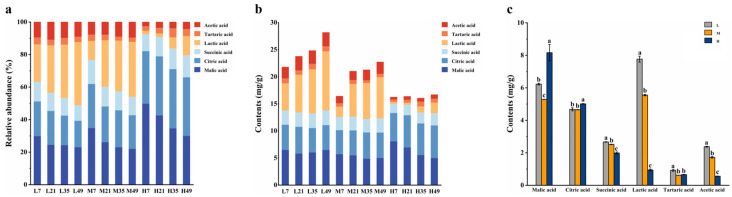
OAs during fermentation of BBP-meju with different salt concentrations: (**a**) content of OAs; (**b**) relative abundance of OAs; (**c**) average content of OAs in BBP-meju samples with different salt concentrations. Where letters a, b, and c indicated the significant differences (*p* < 0.05).

OAs are closely related to the flavor and nutrition of various fermented foods and have been shown to play an essential role in BBP-meju fermentation [44]. The dynamic changes in malic acid, citric acid, succinic acid, lactic acid, tartaric acid, and acetic acid during BBP-meju fermentation with different salt concentrations are shown in Figure 3. The levels of total OAs in BBP-meju with different salt concentrations increased and reached 28.17 ± 1.43 mg/g (L), 22.72 ± 0.18 mg/g (M), and 16.69 ± 1.02 mg/g (H), respectively, on day 49 (Figure 3b). During the fermentation, the levels of total OAs in BBP-meju increased with salt reduction (Figure 3b). This result was consistent with the study by Li et al. [4]. Specifically, malic acid, citric acid, and succinic acid were the major OAs in high-salt meju. In contrast, malic acid, lactic acid, and citric acid were the major OAs in medium- and low-salt meju (Figure 3a). Malic acid, citric acid, and succinic acid were known to be key acidulants in BBP fermentation, imparting a natural sour flavor to BBP and inhibiting the growth of undesirable microorganisms, thereby prolonging the shelf life [45]. The average content of lactic acid was 7.77 ± 0.18 mg/g (L), 5.55 ± 0.04 mg/g (M), and 0.95 ± 0.05 mg/g (H) at the three salt concentrations, showing significant variation with salt concentration (Figure 3c). Meanwhile, the average content of acetic acid increased significantly from 0.56 ± 0.01 mg/g under high-salt conditions to 2.38 ± 0.03 mg/g under low-salt conditions (Figure 3c). *Lactobacillus* has been reported to convert oxaloacetate and pyruvate to acetic or lactic acid [4]. Therefore, the high levels of lactic acid and acetic acid in low-salt meju may be related to the accumulation of *Lactobacillus* in the fermentation system.

Based on these results, FAAs and OAs exhibited continuous changes during the BBP-meju fermentation. Hence, the samples from days 7 and 35 were further selected for subsequent analysis.

### 3.4. Analysis of E-Nose Response during BBP-Meju Fermentation with Different Salt Concentrations

**Figure 4:**
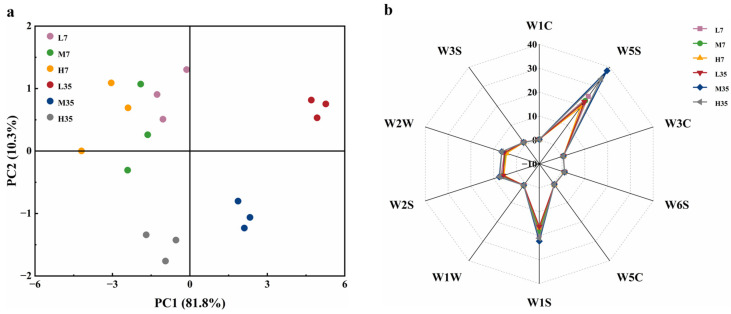
The volatile compounds of BBP-meju with different salt concentrations based on E-nose data: (**a**) PCA scores and (**b**) radar chart.

The E-nose can acquire the flavor characteristics of the sample to be tested by simulating the human olfactory system. Thus, it can avoid the subjectivity of sensory evaluation and is widely used in flavor identification [46]. In this study, an E-nose was used to analyze the flavor characteristics of BBP-meju during fermentation with different salt concentrations. Subtle differences in flavor compounds were captured by differences in sensor response values. Principal component analysis (PCA) was first conducted on the E-nose data, and the results are shown in Figure 4. The results indicated that PC1 and PC2 contributed 10.3% and 81.8% of the variance, respectively, explaining 92.1% of the total variance. Therefore, these two principal components were sufficient to reflect the flavor response information of different sensors for different samples [47]. According to the PCA score plot, samples L35, M35, and H35 were distributed in the first, fourth, and third quadrants, respectively. However, all three samples on day 7 were distributed in the second quadrant (Figure 4a). On day 7, the flavor characteristics of the three samples with different salt concentrations were similar. As the fermentation progressed, the flavor differences among meju samples with different salt concentrations became more pronounced.

The flavor profiles of the different meju samples were further elucidated more intuitively and clearly by radar charts. Overall, the radar chart profiles of all meju samples were relatively similar. However, there were differences in the response of each sensor for different samples (Figure 4b). Sensors W1C, W3C, W6S, W5C, W1W, and W3S showed weak responses to all the samples, while sensors W5S, W1S, W2S, and W2W had strong responses. As fermentation progressed, sensors W5S, W1S, W2S, and W2W significantly enhanced in response to meju samples, and their responses decreased with increasing salt concentration. The results indicated that the various flavor compounds in meju continuously accumulated during fermentation, ultimately forming the rich aroma of BBP-meju. Additionally, salt-reduced fermentation could further increase the level of the flavor compounds, enhancing the characteristic flavor of BBP-meju.

### 3.5. Volatile Flavor Profile during BBP-Meju Fermentation with Different Salt Concentrations

**Figure 5:**
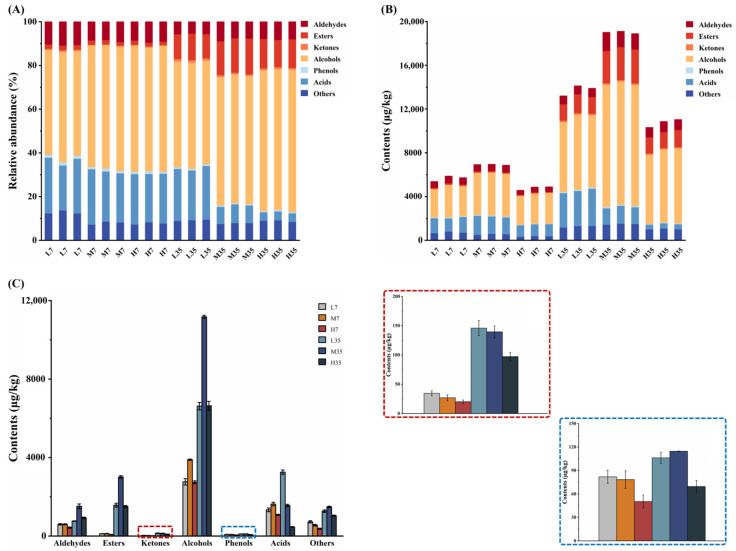
Content distribution of volatile flavor compounds during fermentation of BBP-meju with different salt concentrations: (**A**) relative abundance of different types of volatile flavor compounds; (**B**) content of different types of volatile flavor compounds; (**C**) variation in the content of different types of volatile flavor compounds across samples.

Volatile flavor compounds play a crucial role in determining the flavor and texture of fermented foods and are essential for their quality and consumer acceptance [48]. To further elucidate the differences in flavor compounds during the fermentation of BBP-meju with different salt concentrations, HS-SPME-GC × GC-MS was employed for detection and analysis in this study. The results showed that a total of 66 volatile flavor compounds were identified, which could be classified into 7 major groups, including 11 aldehydes, 12 esters, 4 ketones, 22 alcohols, 5 phenols, 6 acids, and 6 other compounds (Table 2). The relative abundance of the seven groups of volatile flavor compounds in different meju samples, as well as the changes in their contents, are statistically presented in Figure 5. The total amount of volatile flavor compounds increased significantly during BBP-meju fermentation, with increases of 143.19% ± 4.14% (L), 174.94% ± 0.23% (M), and 124.90% ± 1.11% (H), respectively (Figure 5B). Alcohols, esters, aldehydes, and acids consistently remained the main volatile flavor compounds in BBP-meju, and alcohols were always absolutely dominant (Figure 5A). At the three different salt concentrations, the medium-salt (8%) meju had the highest content of volatile flavor compounds, while the high-salt (12%) meju had the lowest content (*p* < 0.05) (Figure 5B). This result differed from the reported by Yang et al. [23]. Specifically, the content of alcohols, acids, esters, ketones, and phenols in low- and medium-salt meju was higher than that in high-salt meju (Figure 5B). This result agreed with the study of Li et al. [4].

Alcohols in fermented foods originate from the metabolism of sugars and amino acids during microbial fermentation. They are essential components that determine the flavor characteristics of BBP [49]. Compared to fermentation on day 7, the alcohol content in meju samples on day 35 significantly increased from 2673.02 ± 158.77 μg/kg (L), 3981.63 ± 32.83 μg/kg (M), and 2937.76 ± 74.01 μg/kg (H) to 6620.24 ± 189.14 μg/kg (L), 11,175.72 ± 75.63 μg/kg (M), and 6637.19 ± 226.84 μg/kg (H), respectively (Table 2, Figure 5B). 3-Methyl-1-butanol, 1-nonanol, 1-octen-3-ol, heptaethylene glycol, and glycerol were the most abundant alcohols in the meju samples on day 35 (Table 2). Among them, 3-methyl-1-butanol and 1-octen-3-ol were the key aroma-active compounds in BBP [48]. By day 35, the contents in the three different meju were medium-salt > low-salt > high-salt. This result indicated that high-salt conditions inhibited the microbial growth associated with forming these two flavor compounds. 3-Methyl-1-butanol has a fatty aroma, while 1-octen-3-ol has a mushroom and fruity odor. Therefore, salt-reduced fermentation may contribute to the accumulation of these two flavors in BBP-meju. However, 2,3-butanediol has intense creamy and fruity aromas, which increase with increasing salt concentration. It was shown that a high-salt environment contributed to the formation and accumulation of specific high-quality flavors.

Ethanol and fatty acids produced by microbial metabolism undergo esterification to generate ethyl acetate. Therefore, the higher level of ethanol in BBP-meju could explain the abundance of ethyl esters [40]. On the other hand, the esterification of fatty alcohols with acetic acid or the alcoholysis pathway with acetyl-CoA can generate acetate esters [50]. High levels of ethyl esters and acetate esters were detected in BBP-meju samples, including ethyl acetate, ethyl palmitate, ethyl phenylacetate, phenethyl acetate, ethyl isovalerate, isobutyl acetate, and isoamyl acetate (Table 2). These compounds together brought fruity, sweet, and floral flavors to BBP-meju. Additionally, they could mask the bitter and pungent odors due to the accumulation of fatty acid and amino acid [51]. Among them, ethyl acetate was not detected on day 7. By day 35, higher levels of ethyl acetate were detected in both medium- and low-salt meju samples at 4.04 ± 0.86 μg/kg (M) and 115.82 ± 4.42 μg/kg (L), respectively, while still not detected in high-salt meju (Table 2). These results indicated that the gradual formation and accumulation of ethyl acetate accompanied the fermentation of BBP-meju. However, the high-salt environment inhibited the activity of the relevant microorganisms, thereby suppressing the accumulation of ethyl acetate in high-salt meju. In addition, ethyl phenylacetate, ethyl isovalerate, isobutyl acetate, isoamyl acetate, and ethyl palmitate all had the highest content in medium-salt meju (Table 2), characterizing the promotion of specific salinity on the activity of associated microorganisms.

Acids and phenols have been reported to be the dominant flavor compounds in low-salt fermented BBP [4]. During fermentation, microorganisms degrade peptides, amino acids, and sugars in raw materials, converting them to pyruvate. Pyruvate is a critical intermediate in organic acid metabolism, further transforming into a large number of volatile organic acids, thereby significantly impacting the flavor of BBP [39]. By day 35, acetic acid and 3-methylbutanoic acid levels were higher in both low- and medium-salt meju than in high-salt meju (Table 2). Acetic acid and 3-methylbutanoic acid both have a sour taste and an unpleasant pungent odor. Moreover, 3-methylbutanoic acid is a typical sour and sweaty compound in BBP [48], affecting the taste and flavor of the final product. Similarly, the levels of phenol and 2,4-di-t-butylphenol in low- and medium-salt meju were significantly higher than in high-salt meju (Table 2), enhancing the pungent odors of BBP-meju. However, 4-hydroxy-3-methoxystyrene and 1-naphthalenol have sweet, floral, and smoky flavors. They were found in the highest contents in low- and medium-salt meju, respectively (Table 2), contributing to the high-quality flavor of low-salt fermented BBP-meju.

It is known that aldehydes and ketones have lower odor thresholds, significantly influencing the flavor characteristics of fermented foods [52]. Benzaldehyde, the most predominant aldehyde in BBP-meju, accumulated rapidly during fermentation and reached a concentration of 375.42 ± 26.64 μg/kg (L), 901.52 ± 142.5 μg/kg (M), and 590.74 ± 18.71 μg/kg (H), respectively, on day 35 (Table 2). It was evident that the benzaldehyde in high-salt meju was significantly lower than that in medium-salt meju (*p* < 0.05) (Table 2), consistent with the studies of Li et al. [4]. Additionally, phenylacetaldehyde, 2-phenyl-2-butenal, and hexanal had the highest abundance in medium-salt meju, exceeding 100 μg/kg (Table 2). They could impart sweet, honey, and fruity flavors to BBP-meju, positively contributing to the flavor of the final product. 3-Octanone was the major ketone in BBP-meju, with its content increasing as salt concentration decreased (Table 2), imparting sweet and fruity flavors to low-salt fermented BBP-meju.

To identify the significantly different volatile flavor compounds during the fermentation of BBP-meju samples with different salt concentrations, partial least squares discriminant analysis (PLS-DA) was conducted, as shown in Figure S1. The values of R2X, Q2Y, and R2Y all exceeded 0.90, indicating the reliability of the PLS-DA model. According to the results, the different salt concentrations of BBP-meju samples exhibited good repeatability, with significant differences among samples on day 35 but no significant differences among samples on day 7 (Figure S1a). This result was in agreement with the results of the E-nose analysis. Furthermore, based on the threshold (i.e., *p* < 0.05 and variable important in projection (VIP) > 1), 22 volatile flavor compounds were selected, including 9 alcohols, 5 aldehydes, 3 phenols, 2 esters, 1 acid, 1 ketone, and 1 ether (Figure S1b). They were considered to play a key role in differentiating the flavor of low-salt fermented BBP-meju.

### 3.6. Analysis of Microbial Community during Fermentation of BBP-Meju with Different Salt Concentrations

#### 3.6.1. Analysis of Abundance of Microbial Community

To elucidate the effect of salt-reduced fermentation on the microbial community during BBP-meju fermentation, 18 meju samples with different salt concentrations were subjected to high-throughput sequencing using the Illumina NovaSeq platform. After quality filtering, primer sequence identification and removal, assembly, and removal of chimeras, the high-quality sequences for bacteria and fungi were 37,236–70,391 and 61,232–77,852, respectively (Table 3). They accounted for 80.27–91.45% and 95.04–97.50% of the effective sequences, respectively (Table 3). The Shannon index curve and rarefaction curve can reflect the microbial diversity of each sample at different sequencing depths and indirectly indicate species abundance. A flat curve indicates sufficient sequencing depth where the feature abundance does not increase with additional sequencing. In contrast, a non-flat curve indicates unsaturation, suggesting that increasing data volume may reveal more features [53]. The results showed that the Shannon index curve and the rarefaction curve flattened for the different salt concentrations of BBP-meju, indicating that the sequencing depth could characterize the microbial diversity within the samples (Figure S2).

**Figure 6:**
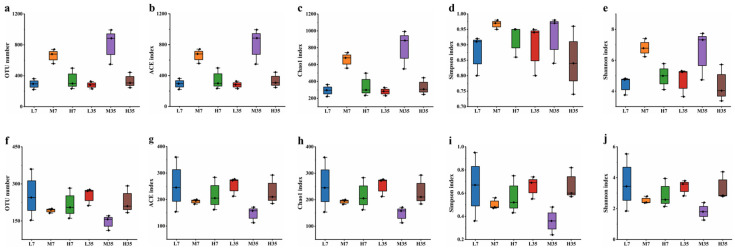
The α-diversity of bacterial (**a**–**e**) and fungal (**f**–**j**) communities during fermentation of BBP-meju with different salt concentrations.

At a similarity level of 97% using USEARCH, sequences were clustered. OTUs were filtered at a threshold of 0.005% of all sequences, resulting in a total of 8148 bacterial OTUs and 3870 fungal OTUs (Table S1). The coverage of all BBP-meju samples exceeded 99.90%, indicating that almost all sequences in the samples were detected (Table S1). This result could effectively reflect the microbial community characteristics of BBP-meju samples with different salt concentrations (Table S1 and Figure 6). The species abundance of microbial communities in BBP-meju samples with different salt concentrations was further characterized by the ACE index and Chao1 index. Meanwhile, the Simpson and Shannon indices were utilized to characterize species diversity [54]. The results indicated that bacterial OTUs were greater than fungal OTUs in all meju samples, suggesting a higher abundance of bacteria than fungi, consistent with previous reports [23]. During the fermentation, the abundance and diversity of bacterial communities in medium-salt meju were consistently higher than those in low- and high-salt meju (Figure 6b–e). In contrast, the highest abundance and diversity of fungal communities were found in low-salt meju, followed by high-salt meju, and the lowest in medium-salt meju (Figure 6g–j). These findings suggested that moderate salt-reduced fermentation contributed to the proliferation and succession of bacterial and fungal communities in BBP-meju. However, deficient salt levels could lead to dysbiosis of the internal flora of the meju, resulting in reduced levels of abundance and diversity.

#### 3.6.2. Analysis of Microbial Community Structure

**Figure 7:**
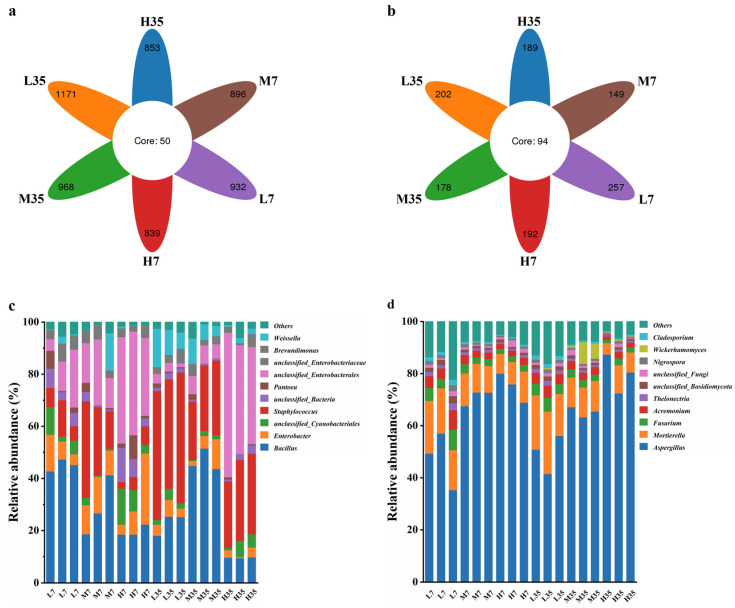
Structural analysis of bacterial and fungal communities in BBP-meju samples: (**a**,**b**) comparison of OTUs of bacteria (**a**) and fungi (**b**); (**c**,**d**) relative abundance of bacteria (**c**) and fungi (**d**) at the genus level.

Differential analysis of OTUs for BBP-meju samples with different salt concentrations was conducted, and the results are shown in Figure 7. It could be seen that the unique bacterial OTU numbers in L7, M7, H7, L35, M35, and H35 samples were 932, 896, 839, 1171, 968, and 853, respectively, while the shared bacterial OTU number was 50 (Figure 7a). Relative to fungi, the number of unique fungal OTUs in each BBP-meju sample was notably lower than in bacteria. However, the shared fungal OTU number was 94 (Figure 7b), significantly higher than the bacterial community. This result suggested a higher similarity in the fungal community during the fermentation with different salt concentrations. Nevertheless, the overall abundance of the bacterial community was significantly higher than the fungal community, with more pronounced differentiation across different salt concentrations.

According to the histograms of the distribution of bacterial communities at genus level during BBP-meju fermentation, the top 10 relatively abundant bacterial genera were *Bacillus*, *Enterobacter*, *unclassified_Cyanobacteriales*, *Staphylococcus*, *unclassified_Bacteria*, *Pantoea*, *unclassified_Enterobacterales*, *unclassified_Enterobacteriaceae*, *Brevundimonas*, and *Weissella* (Figure 7c). Among them, *Bacillus* was the dominant bacterial community in all BBP-meju samples (Figure 7c). On day 7, *Bacillus* exhibited the highest relative abundance in low-salt meju, ranging from 42.76% to 47.34%, whereas its highest abundance was reached in medium-salt meju on day 35 (43.66–51.51%) (Figure 7c). *Bacillus* was reported to be a functional core microbial community during BBP fermentation. *Bacillus* secreted abundant proteolytic enzymes, leading to the accumulation of flavor compounds, such as amino acids, crucial for the distinctive flavor formation of BBP [49]. Thus, the high abundance of *Bacillus* on day 7 may explain the rapid increase in AAN levels in early-stage fermentation. The decrease in *Bacillus* abundance on day 35 corresponded to the decline in AAN levels in middle and late fermentation. Meanwhile, the low-salt conditions could alleviate the osmotic stress of salt concentration on microorganisms and their secreted proteases, which caused the elevation of amino acid levels in low-salt meju. Aside from *Bacillus*, *Staphylococcus* is also a core dominant microbiota during BBP fermentation, which is one of the key factors influencing the flavor and quality of the BBP [55]. The relative abundance of *Staphylococcus* in BBP-meju increased significantly as fermentation progressed. On day 7, the relative abundance of *Staphylococcus* in medium-salt meju ranged from 14.32% to 36.92%, the highest among the three salt concentrations. However, by day 35, the relative abundance of *Staphylococcus* in low-salt meju reached 41.93–50.21%, significantly higher than that in medium- and high-salt meju (*p* < 0.05) (Figure 7c). It is known that the growth of *Staphylococcus* can cause the accumulation of large amounts of acids during BBP fermentation [56]. This explained the consistency of the differential succession of *Staphylococcus* in BBP-meju at different salt concentrations with the trend of acid substances shown in Figure 5. Notably, three opportunistic pathogens, *Enterobacter*, *Pantoea*, and *Brevundimonas*, were also found among these genera. The relative abundance of all three bacterial genera showed a decreasing trend during BBP-meju fermentation and further decreased with increasing salt concentration (Figure 7c). This result suggested that high-salt conditions could effectively inhibit the propagation of pathogenic microorganisms. However, the salt-reduced fermentation of BBP will provide a favorable environment for the growth of opportunistic pathogens, posing a threat to human health and increasing the food safety risks of BBP products. To address this issue, researchers proposed inoculating competitive starter cultures, especially indigenous microorganisms, to antagonize opportunistic pathogens in low-salt fermented foods, thereby enhancing product quality. Zang et al. [57] fermented Suanyu using a mixed starter culture consisting of *Lactobacillus plantarum* 120, *Staphylococcus xylosus* 135, and *Saccharomyces cerevisiae* 31. It was found that adding this mixed starter culture could significantly enhance the sensory characteristics of Suanyu and effectively inhibit the growth of spoilage microorganisms. Furthermore, the addition of indigenous yeasts *Torulaspora delbrueckii* and *Pichia guilliermond* during low-salt fermentation of soy sauce could effectively inhibit the growth of *Staphylococcus* and *Enterococcus* spp. while producing a richer flavor profile, thus improving the flavor impact caused by reduced-salt fermentation [58].

In terms of fungi, the 10 fungal genera with the highest relative abundance were Aspergillus, Mortierella, Fusarium, Acremonium, Thelonectria, unclassified_Basidiomycota, unclassified_Fungi, Nigrospor, Wickerhamomyces, and Cladosporium. Aspergillus and Mortierella constituted the dominant fungal communities in all BBP-meju samples (Figure 7d). The high levels of Aspergillus in BBP-meju were mainly due to its use as a starter for koji production. Aspergillus is known to secrete amylases, proteases, and other enzymes, leading to the formation and accumulation of numerous flavor compounds during BBP fermentation, while also creating suitable conditions for the growth of other microorganisms in the later stages of fermentation [59]. The results showed that the relative abundance of Aspergillus was relatively stable during fermentation, reaching 41.51–56.11% (L), 63.25–67.16% (M), and 72.49–87.42% (M), respectively, by day 35 (Figure 7d). This indicated that Aspergillus demonstrated good adaptability to the high-salt environment in BBP-meju. However, the metabolism of Aspergillus is closely linked to the development of musty and metallic off-flavors in BBP, along with the presence of mycelium in the final product, consequently affecting consumer acceptance [60]. Mortierella is capable of producing polyunsaturated fatty acids and exhibits inhibitory effects on some pathogens [61]. The relative abundance of Mortierella did not change significantly during fermentation, but decreased significantly with increasing salt concentrations, in contrast to Aspergillus (Figure 7d). This suggested that the high osmotic pressure under high-salt conditions exerted inhibitory effects on the growth of Mortierella, while salt-reduced fermentation provided suitable conditions for the accumulation of flavor compounds in BBP-meju. This result was consistent with the report of Yang et al. [23].

*Bacillus*, *Staphylococcus*, *Aspergillus*, and *Mortierella* were the predominant microbial communities during BBP-meju fermentation. Salt reduction promoted the growth of *Bacillus*, *Staphylococcus*, and *Mortierella* while inhibiting the growth of *Aspergillus*. Therefore, salt-reduced fermentation facilitated the production of flavor compounds, such as amino acids and organic acids, while attenuating the musty taste of *Aspergillus*.

### 3.7. Correlation of Microbial Community with Volatile Flavor Compounds

**Figure 8:**
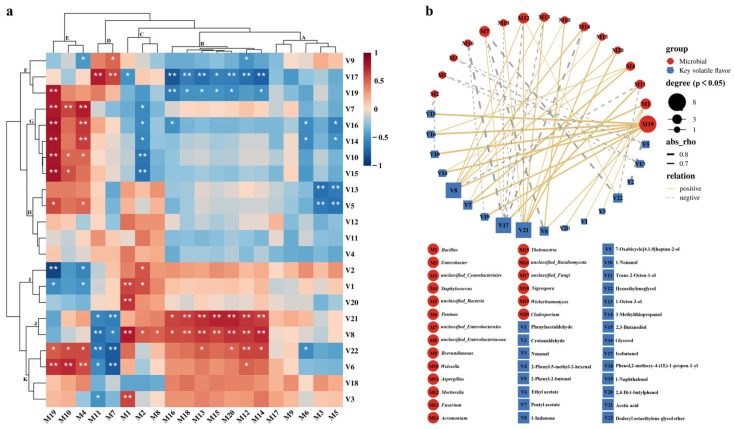
Correlation clustering heat map (**a**) and correlation network diagram (**b**) between dominant microbial genera and key volatiles flavor compounds (VIP > 1) during BBP-meju fermentation with different salt concentrations. (**a**) The red and blue indicate positive and negative correlation, respectively, and the darker the color, the stronger the correlation; asterisks represent significance, * is *p* < 0.05, and ** is *p* < 0.01. (**b**): blue squares represent key volatile flavor compounds, red circles represent dominant microbial genera, and the yellow solid line and gray dotted line represent positive and negative correlation, respectively, and the thicker the lines, the stronger the correlation.

Based on the Spearman correlation coefficient, correlation clustering heatmaps and correlation network diagrams were used to reveal the potential correlations between dominant microbial communities and 22 key volatile flavor compounds with *p* < 0.05 and VIP > 1, aiming to capture the core microbial community in the salt-reduced fermentation. The results showed that the 20 dominant microbial genera could be classified into five categories, A, B, C, D, and E, while the 22 key volatile flavor compounds could be classified into six categories, F, G, H, I, J, and K (Figure 8a). The microbial genera of class B were significantly negatively correlated with isobutanol (V17) and 1-naphthalenol (V19) in class F flavor compounds, but significantly positively correlated with the flavor compounds of class J; microbial genera of class C showed a significant positive correlation with the flavor compounds of classes I and J; however, *Enterobacter* (M2) in this group exhibited a significant negative correlation with the flavor compounds of class G; the microbial genera of class D were significantly negatively correlated with the flavor compounds of class J and K, but showed a significant positive correlation with isobutanol (V17); microbial genera of class E were significantly positively correlated with flavor compounds of classes G and K (Figure 8a). Furthermore, according to the correlation network diagram, 16 dominant microbial genera were significantly correlated with 17 key volatile flavor compounds (*p* < 0.01, |ρ| > 0.6) (Figure 8b). Among them, *Wickerhamomyces* (M19), *Bacillus* (M1), *Staphylococcus* (M4), and *Mortierella* (M12) all showed highly significant positive correlations with ≥3 volatile flavor compounds. They may play crucial roles in forming characteristic flavors in different salt concentrations of BBP-meju [62]. These results agreed with the high-throughput sequencing results, again emphasizing the importance of *Bacillus*, *Staphylococcus*, and *Mortierella*. *Wickerhamomyces* (M19) is a dominant fungal community in traditional fermented foods, such as kimchi, sour pork, and sourdough. It can secrete proteases that hydrolyze proteins in raw materials into peptides and amino acids, further converting them into various flavor compounds. Therefore, *Wickerhamomyces* (M19) can impart freshness to the products and coordinate the overall aroma characteristics of fermented foods [63,64]. Research by Chen et al. [65] indicated that *Wickerhamomyces* (M19) was positively correlated with esters such as ethyl acetate, heptadecanoic acid ethyl ester, ethyl tridecanoate, and decanoic acid ethyl ester, especially a high-quality producer of ethyl acetate [66]. In this study, *Wickerhamomyces* (M19) showed significantly positive correlations with ethyl acetate (V6), pentyl acetate (V7), 1-nonanol (V10), 3-methylthiopropanol (V14), 2,3-butanediol (V15), glycerol (V16), and 1-naphthalenol (V19). However, it negatively correlated significantly with crotonaldehyde (V2) (Figure 8b). Meanwhile, the relative abundance of *Wickerhamomyces* (M19) increased gradually during fermentation, with the highest abundance in medium-salt meju and the lowest in low-salt meju (Figure 8b). It is known that *Wickerhamomyces* (M19) has a good tolerance to high osmotic pressure [67]. Therefore, the high abundance of *Wickerhamomyces* (M19) in medium-salt meju contributed to the accumulation of fruity, sweet, and other quality flavors and attenuated the harsh flavors caused by crotonaldehyde (V2). Apart from *Wickerhamomyces* (M19), *Staphylococcus* (M4) and *Weissella* (M10) were significantly positively correlated with ethyl acetate (V6) and pentyl acetate (V7) (Figure 8b). This suggested a synergistic effect among *Wickerhamomyces* (M19), *Staphylococcus* (M4), and *Weissella* (M10), collectively promoting the formation of ester flavors. At day 35 of fermentation, according to Figure 7c, the relative abundances of *Staphylococcus* (M4) and *Weissella* (M10) increased with salt reduction. This indicated that salt-reduced fermentation promoted the growth of microbial genera associated with the synthesis of ester flavor compounds, thereby contributing positively to the overall flavor of BBP-meju. Similarly, within microbial class B, *Mortierella* (M12), *Fusarium* (M13), *Acremonium* (M14), *Thelonectria* (M15), *Nigrospora* (M18), and *Cladosporium* (M20) were all significantly positively correlated with 1-indanone (V8) and acetic acid (V21), while showing a significant negative correlation with isobutanol (V17) (Figure 8b). By day 35, the relative abundance of all the above microbial genera increased with salt reduction (Figure 7c). This result suggested that salt reduction could alleviate the inhibition of their growth by high osmotic pressure, further promoting the accumulation of acidic flavors through synergistic effects and inhibiting off-flavors produced by isobutanol (V17). As the absolute dominant bacterial genus in all meju samples, *Bacillus* (M1) showed a highly significant positive correlation with phenylacetaldehyde (V1), nonanal (V3), 1-indanone (V8), and 2,4-di-t-butylphenol (V20), while showing a significant negative correlation with isobutanol (V17) (*p* < 0.05) (Figure 8b). This result aligned with the positive correlation of *Bacillus* with most of the high-quality volatile flavor compounds reported by Yang et al. [23]. Meanwhile, *Bacillus* (M1) could, to some extent, suppress the production of the unpleasant compound isobutanol (V17), further facilitating the formation of high-quality flavors in BBP-meju during salt-reduced fermentation. Notably, as a dominant fungal community in the fermentation of BBP-meju, *Aspergillus* (M11) was significantly negatively correlated with nonanal (V3), ethyl acetate (V6), 1-indanone (V20), and acetic acid (V21) while showing a significant positive correlation with isobutanol (V17) (Figure 8b). Thus, high levels of *Aspergillus* (M11) in high-salt meju would be detrimental to the formation and accumulation of high-quality flavor compounds.

In conclusion, the key microbial genera *Bacillus*, *Staphylococcus*, and *Mortierella* in BBP-meju all play critical roles in forming high-quality volatile flavor compounds. They may be the core functional microbiota in the fermentation process of BBP-meju, which is essential for forming its characteristic flavors. Furthermore, salt-reduced fermentation further accumulated high-quality flavor compounds in BBP-meju, positively impacting its characteristic flavor development.

## 4. Conclusions

This study comprehensively investigated the physicochemical properties, volatile flavor compounds, and microbial community dynamics during BBP-meju fermentation with different salt concentrations. Further analysis was conducted on the correlation between the dominant microbial communities and key volatile flavor compounds. The results indicated that the salt reduction contributed to the accumulation of AAN, reducing sugars, FAAs, OAs, and volatile flavor compounds during BBP-meju fermentation, while also increasing the total acid level. High-throughput sequencing results revealed that *Bacillus*, *Staphylococcus*, *Aspergillus*, and *Mortierella* were the dominant microbial communities during fermentation. Except for *Aspergillus*, their relative abundance was elevated in salt-reduced fermentation and positively correlated with several high-quality flavor compounds. However, the presence of higher levels of opportunistic pathogens in the low-salt meju posed a threat to the safety of the final product. These results provided a theoretical basis for the quality and safety control of BBP during salt-reduced fermentation, as well as a guideline for further developing the low-salt fermented food industry.

## Figures and Tables

**Table 1:** The sensitivities of sensors contained in the PEN3 electronic nose sensor array.

Sensor Number	Sensor Name	Performance Description
1	W1C	Aromatic compounds
2	W5S	Broad range, react on nitrogen oxides
3	W3C	Ammonia, aromatic compounds
4	W6S	Hydrocarbons
5	W5C	Alkanes, aromatic compounds
6	W1S	Methane, broad range of compounds
7	W1W	Sulfur compounds, terpenes
8	W2S	Broad range, alcohols
9	W2W	Organic sulfur compounds
10	W3S	Methane, aliphatic compounds

**Table 2:** Relative contents (μg/kg) of volatile compounds during fermentation of BBP-meju with different salt concentrations.

Compounds	CAS N.	RI	MW	L7	M7	H7	L35	M35	H35
Aldehydes									
Benzaldehyde	100-52-7	1520	106	211.20 ± 25.03	202.72 ± 31.54	105.28 ± 6.93	375.42 ± 26.64	901.52 ± 142.50	590.74 ± 18.71
Apricolin	104-61-0	2024	156	5.64 ± 1.15	7.83 ± 1.50	4.15 ± 0.37	8.64 ± 1.39	13.08 ± 2.51	8.01 ± 0.96
Valeraldehyde	110-62-3	979	86	21.04 ± 2.31	20.42 ± 1.24	23.94 ± 2.85	n.d.	n.d.	n.d.
Phenylacetaldehyde	122-78-1	1641	120	184.42 ± 37.72	165.04 ± 16	103.33 ± 14.12	62.40 ± 7.80	123.84 ± 15.01	78.05 ± 6.01
Crotonaldehyde	123-73-9	1039	70	12.96 ± 2.58	9.97 ± 1.44	4.77 ± 0.34	n.d.	n.d.	n.d.
Nonanal	124-19-6	1391	142	18.64 ± 2.42	5.52 ± 0.65	5.97 ± 0.60	6.48 ± 0.64	10.92 ± 1.50	7.00 ± 0.93
2-Phenyl-5-methyl-2-hexenal	21834-92-4	2056	188	3.14 ± 0.36	4.18 ± 0.27	1.91 ± 0.39	0.86 ± 0.06	4.39 ± 0.59	3.29 ± 0.33
2-Phenyl-2-butenal	4411-89-6	1925	146	1.99 ± 0.53	46.13 ± 4.22	18.71 ± 2.08	41.02 ± 3.72	78.61 ± 3.39	37.57 ± 1.38
2-Heptenal	57266-86-1	1322	112	62.46 ± 27.04	54.47 ± 8.83	73.33 ± 7.26	127.99 ± 9.04	197.97 ± 7.72	71.04 ± 4.18
Hexanal	66-25-1	1083	100	77.79 ± 22.37	83.92 ± 4.55	90.47 ± 6.70	129.98 ± 8.11	177.17 ± 7.27	125.81 ± 17.98
5-Hydroxymethylfurfural	67-47-0	2496	126	n.d.	n.d.	n.d.	10.31 ± 1.34	11.90 ± 2.54	7.61 ± 0.58
Esters									
Ethyl phenylacetate	101-97-3	1783	164	7.71 ± 1.34	4.30 ± 0.30	2.13 ± 0.91	12.83 ± 0.24	27.91 ± 2.48	12.99 ± 2.23
Phenethyl acetate	103-45-7	1813	164	30.33 ± 2.36	17.33 ± 4.73	8.01 ± 0.71	59.10 ± 7.29	171.31 ± 4.41	97.81 ± 14.24
Ethyl isovalerate	108-64-5	1068	130	21.34 ± 3.94	33.77 ± 7.24	32.74 ± 6.23	114.48 ± 14.71	194.59 ± 5.08	74.18 ± 6.29
Isobutyl acetate	110-19-0	1012	116	n.d.	n.d.	n.d.	49.14 ± 5.77	85.72 ± 6.25	36.79 ± 2.29
Isoamyl acetate	123-92-2	1123	130	33.55 ± 4.54	42.07 ± 8.34	26.73 ± 3.94	1105.69 ± 69.78	2318.01 ± 50.17	1214.85 ± 36.47
Ethyl acetate	141-78-6	888	88	n.d.	n.d.	n.d.	115.82 ± 4.42	4.04 ± 0.86	n.d.
Hexyl acetate	142-92-7	1273	144	n.d.	n.d.	n.d.	12.54 ± 1.81	5.97 ± 1.79	4.76 ± 0.60
3-Hydroxydihydro-2(3H)-furanone	19444-84-9	2142	102	2.89 ± 0.27	7.27 ± 0.65	2.65 ± 0.37	8.96 ± 1.06	19.77 ± 4.84	13.33 ± 3.04
Pentyl acetate	628-63-7	1176	130	n.d.	n.d.	n.d.	29.56 ± 5.74	4.95 ± 0.84	20.61 ± 3.79
Ethyl palmitate	628-97-7	2251	284	20.02 ± 3.69	22.43 ± 6.14	8.70 ± 0.75	22.59 ± 2.25	141.25 ± 10.07	19.42 ± 2.13
Isoamyl isovalerate	659-70-1	1293	172	n.d.	n.d.	n.d.	26.40 ± 1.51	26.07 ± 3.15	9.30 ± 1.10
Oct-1-en-1-yl acetate	77149-68-9	-	170	n.d.	n.d.	n.d.	18.99 ± 3.29	13.06 ± 1.52	4.14 ± 0.78
Ketones									
3-Octanone	106-68-3	1253	128	n.d.	n.d.	n.d.	107.83 ± 13.27	100.55 ± 8.66	66.61 ± 4.30
6-Methylhept-5-en-2-one	110-93-0	1339	126	5.30 ± 0.61	7.51 ± 0.97	4.81 ± 0.56	6.03 ± 0.78	10.69 ± 2.43	6.10 ± 1.01
2-Octanone	111-13-7	1287	128	18.48 ± 4.47	15.09 ± 5.06	13.68 ± 2.29	29.07 ± 5.59	25.06 ± 3.17	24.57 ± 4.55
1-Indanone	83-33-0	1969	132	10.80 ± 1.20	4.31 ± 1.17	1.71 ± 0.17	2.98 ± 0.24	3.27 ± 0.56	n.d.
Alcohols									
2-Ethylhexanol	104-76-7	1491	130	2.64 ± 0.36	2.31 ± 0.65	1.99 ± 0.44	4.36 ± 0.87	2.16 ± 0.76	1.22 ± 0.35
7-Oxabicyclo[4.1.0]heptan-2-ol	1192-78-5	-	114	63.57 ± 11.36	53.02 ± 7.55	53.54 ± 6.46	26.54 ± 3.12	50.92 ± 6.88	58.01 ± 4.96
3-Methyl-1-butanol	123-51-3	1209	88	257.77 ± 27.04	493.88 ± 35.04	356.84 ± 32.37	1555.05 ± 124.29	2279.02 ± 23.54	987.25 ± 54.18
1-Nonanol	143-08-8	1660	144	2.11 ± 0.57	n.d.	n.d.	614.12 ± 33.58	1018.33 ± 69.98	1555.05 ± 29.24
4-Phenyl-3-buten-2-ol	17488-65-2	-	148	n.d.	n.d.	n.d.	5.94 ± 1.52	11.41 ± 1.91	5.08 ± 0.97
Trans-2-Octen-1-ol	18409-17-1	1613	128	n.d.	123.7 ± 10.07	79.07 ± 8.03	62.23 ± 7.46	71.87 ± 6.98	56.19 ± 4.73
DL-β-Ethylphenethyl alcohol	2035-94-1	1978	150	n.d.	n.d.	n.d.	9.05 ± 1.38	12.01 ± 2.26	4.88 ± 1.35
2-Nonen-1-ol	22104-79-6	1692	142	n.d.	n.d.	n.d.	12.71 ± 2.43	30.80 ± 1.85	16.21 ± 1.14
Hexaethyleneglycol	2615-15-8	-	282	22.43 ± 4.92	75.40 ± 6.24	84.44 ± 7.47	61.33 ± 13.09	173.39 ± 18.23	12.71 ± 0.94
1-Octen-3-ol	3391-86-4	1450	128	1089.31 ± 80.20	1955.59 ± 88.13	1273.05 ± 57.24	1503.75 ± 104.23	2574.37 ± 90.06	1215.44 ± 66.28
3-Methylthiopropanol	505-10-2	1719	106	25.75 ± 3.18	24.68 ± 4.64	15.07 ± 3.10	105.89 ± 7.47	127.21 ± 2.27	1585.01 ± 15.91
3,6,9,12,15,18,21-Heptaoxatricosane-1,23-diol	5117-19-1	-	370	451.37 ± 23.44	421.89 ± 67.97	277.92 ± 25.86	1257.43 ± 41.33	2234.17 ± 82.86	66.16 ± 3.62
2,3-Butanediol	513-85-9	1543	90	16.22 ± 2.31	11.92 ± 2.01	11.72 ± 2.61	47.86 ± 6.41	269.48 ± 6.66	516.18 ± 19.89
2-Heptanol	543-49-7	1320	116	n.d.	0.67 ± 0.06	0.65 ± 0.15	3.92 ± 1.33	4.98 ± 0.55	2.18 ± 0.50
Heptaethylene glycol	5617-32-3	-	326	750.05 ± 15.98	618.79 ± 27.42	517.64 ± 36.38	1133.89 ± 103.43	1687.66 ± 51.89	1092.49 ± 87.83
Glycerol	56-81-5	2303	92	n.d.	20.35 ± 3.68	n.d.	57.41 ± 6.31	313.04 ± 35.48	712.67 ± 29.45
3-Octyl alcohol	589-98-0	1393	130	20.80 ± 9.84	28.42 ± 4.40	24.21 ± 4.9	49.39 ± 6.73	117.58 ± 6.76	105.33 ± 14.29
Ethanol	64-17-5	932	46	32.05 ± 3.62	13.22 ± 1.70	2.88 ± 0.70	35.72 ± 3.41	117.03 ± 8.52	7.74 ± 1.12
Butanol	71-36-3	1142	74	2.54 ± 0.46	1.91 ± 0.24	2.31 ± 0.72	3.97 ± 1.06	8.01 ± 0.43	6.16 ± 0.81
Pentanol	71-41-0	1250	88	16.09 ± 3.36	15.23 ± 3.16	17.28 ± 3.19	42.04 ± 5.10	28.54 ± 4.10	21.05 ± 2.05
Isobutanol	78-83-1	1092	74	n.d.	16.89 ± 4.27	12.81 ± 2.96	4.38 ± 0.15	9.69 ± 1.25	16.02 ± 1.42
Furfuryl alcohol	98-00-0	1661	98	10.31 ± 1.27	13.74 ± 3.76	6.32 ± 0.56	23.23 ± 4.86	34.04 ± 1.94	20.60 ± 2.27
Phenols									
Phenol	108-95-2	2000	94	37.53 ± 2.95	32.10 ± 5.87	20.88 ± 6.79	88.39 ± 5.72	58.85 ± 3.54	24.00 ± 3.16
Phenol,2-methoxy-4-(1E)-1-propen-1-yl	5932-68-3	2362	164	5.17 ± 0.66	n.d.	0.43 ± 0.05	11.36 ± 2.70	n.d.	6.04 ± 0.64
4-Hydroxy-3-methoxystyrene	7786-61-0	2188	150	4.43 ± 2.34	7.63 ± 1.99	4.24 ± 0.85	1.27 ± 0.07	10.29 ± 1.25	6.50 ± 0.97
1-Naphthalenol	90-15-3	-	144	2.19 ± 0.60	3.03 ± 0.46	3.67 ± 0.46	3.87 ± 0.60	9.51 ± 1.12	12.97 ± 2.35
2,4-Di-t-butylphenol	96-76-4	2321	206	32.58 ± 3.39	35.69 ± 7.33	21.06 ± 1.74	1.27 ± 0.29	36.01 ± 3.30	20.01 ± 1.92
Acids									
Octanoic acid	124-07-2	2060	144	10.17 ± 1.34	14.08 ± 4.26	4.63 ± 1.05	14.57 ± 2.34	32.33 ± 1.29	12.16 ± 1.72
3,6,9-Trioxaundecanedioic acid	13887-98-4	-	222	n.d.	n.d.	n.d.	68.78 ± 10.04	16.22 ± 0.90	6.42 ± 0.67
3-Methylbutanoic acid	503-74-2	1666	102	176.89 ± 15.88	304.65 ± 34.84	199.65 ± 18.91	374.81 ± 21.66	1246.03 ± 45.46	313.80 ± 25.25
Acetic acid	64-19-7	1449	60	1131.94 ± 98.26	1292.95 ± 74.64	863.68 ± 41.96	2765.65 ± 85.66	202.76 ± 39.63	101.72 ± 8.92
Agaric acid	666-99-9	-	416	5.64 ± 1.22	2.37 ± 0.61	2.22 ± 0.51	17.38 ± 1.52	14.99 ± 2.76	7.53 ± 0.98
Isobutyric acid	79-31-2	1570	88	11.49 ± 3.14	17.78 ± 4.31	11.42 ± 1.12	17.52 ± 2.43	42.86 ± 1.66	21.70 ± 3.62
Others									
Styrene	100-42-5	1261	104	n.d.	n.d.	n.d.	162.42 ± 2.41	303.57 ± 16.54	321.14 ± 18.16
2,3-Dihydrofuran	1191-99-7	-	70	11.08 ± 1.21	n.d.	n.d.	12.38 ± 2.30	26.47 ± 6.20	11.16 ± 1.44
12-Crown-4	294-93-9	-	176	n.d.	n.d.	n.d.	103.34 ± 17.03	33.37 ± 2.26	21.69 ± 1.94
Dodecyl octaethylene glycol ether	3055-98-9	-	538	497.12 ± 26.96	439.28 ± 44.62	241.99 ± 23.12	609.81 ± 61.44	572.30 ± 43.08	432.97 ± 18.86
Hexamethylcyclotrisiloxane	541-05-9	-	222	216.53 ± 29.17	116.39 ± 24.08	129.30 ± 5.65	366.79 ± 33.16	497.18 ± 10.58	231.39 ± 10.99
Octadecane,3-ethyl-5-(2-ethylbutyl)	55282-12-7	-	366	2.19 ± 1.57	n.d.	1.33 ± 0.28	12.87 ± 2.08	53.12 ± 5.23	15.97 ± 1.77

MW: molecular weight; RI: retention index; n.d.: volatile compounds not detected.

**Table 3:** OTUs distribution on effective and high-quality sequences number of BBP-meju under different salt concentrations.

Samples	Bacteria			Fungal		
	Effective Sequences	High-Quality Sequences	Proportions (%)	Effective Sequences	High-Quality Sequences	Proportions (%)
L7a	58,786	51,006	86.77	79,408	77,311	97.36
L7b	55,060	48,152	87.45	79,349	76,322	96.19
L7c	53,262	44,432	83.42	64,427	61,232	95.04
M7a	59,931	52,944	88.34	79,834	77,046	96.51
M7b	47,009	37,853	80.52	79,900	77,360	96.82
M7c	49,382	39,638	80.27	79,670	76,885	96.50
H7a	60,083	48,920	81.42	79,347	76,045	95.84
H7b	45,747	37,236	81.40	79,642	77,067	96.77
H7c	75,497	66,553	88.15	79,429	77,070	97.03
L35a	59,664	53,408	89.51	79,848	77,580	97.16
L35b	59,740	50,317	84.23	79,681	76,918	96.53
L35c	53,049	42,808	80.70	79,504	76,834	96.64
M35a	56,810	49,493	87.12	79,866	76,747	96.09
M35b	76,969	70,391	91.45	79,381	76,878	96.85
M35c	76,296	69,324	90.86	79,952	77,843	97.36
H35a	60,505	50,971	84.24	79,845	77,852	97.50
H35b	63,071	50,920	80.73	79,611	77,127	96.88
H35c	68,939	60,942	88.40	79,808	76,747	96.16

## Data Availability

The original contributions presented in the study are included in the article and Supplementary Materials, further inquiries can be directed to the corresponding author.

## Supplementary Materials

The following supporting information can be downloaded at: https://www.mdpi.com/article/10.3390/foods13132108/s1, Figure S1: PLS-DA diagram results of volatile flavor compounds during fermentation of BBP-meju with different salt concentrations; Figure S2: Rarefaction curves and Shannon index curve for bacteria (a,b) and fungi (c,d); Table S1: The α-diversity of the bacterial and fungal community during fermentation of BBP-meju with different salt concentrations.
