# Supplementary material for: Effects of Salinity on Physicochemical Properties, Flavor Compounds, and Bacterial Communities in Broad Bean Paste-Meju Fermentation
Source: Foods. 2024 Jul 2;13(13):2108. doi: 10.3390/foods13132108 (PMC11241834; doi:10.3390/foods13132108)
Supplement: Supplementary file 1 [file foods-13-02108-s001.zip › foods-3062900-supplementary.pdf]

**Supplementary Materials for:**

# Effects of salinity on physicochemical properties, flavor compounds, and bacterial communities in broad bean paste-meju fermentation

**Qingyan Guo [1,2,*], Jiabao Peng [1], Jingjing Zhao [1], Jie Lei [1], Yukun Huang [1,2] and Bing Shao [1,3]**

1 Food Microbiology Key Laboratory of Sichuan Province, School of Food and Bioengineering, Xihua University, Chengdu 610039, China; 19150356036@163.com (J.P.); zhaojingjingly@163.com (J.Z.); 0120210019@mail.xhu.edu.cn (J.L.); hyk_diana@163.com (Y.H.); shaobing@163.com (B.S.)

2 Chongqing Key Laboratory of Speciality Food Co-Built by Sichuan and Chongqing, Chengdu 610039, China

3 Beijing Key Laboratory of Diagnostic and Traceability Technologies for Food Poisoning, Beijing Center for Disease Prevention and Control, Beijing 100013, China

* Correspondence: gg2016@mail.ustc.edu.cn; Tel.: +86-18297944809

# Supplementary Materials

**Supplementary Fig. 1** PLS-DA diagram results of volatile flavor compounds during fermentation of BBP-meju with different salt concentrations. (A) Score scatter plot; (B) VIP plot.

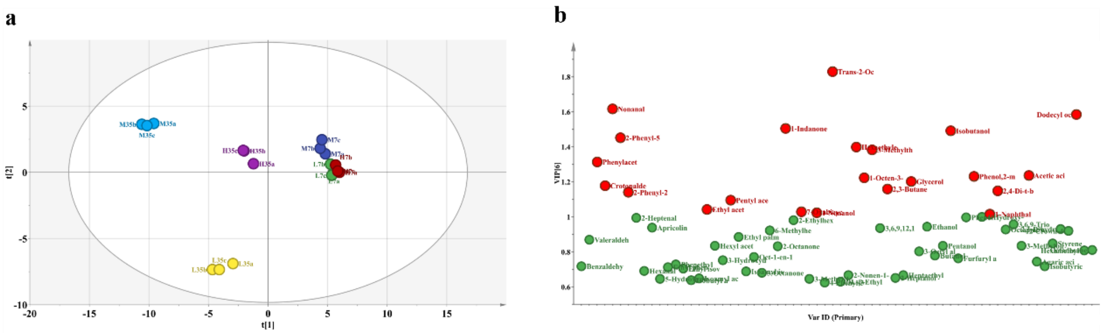

**Supplementary Fig. 2** Rarefaction curves and Shannon index curve for bacteria (a,b) and fungi (c,d).

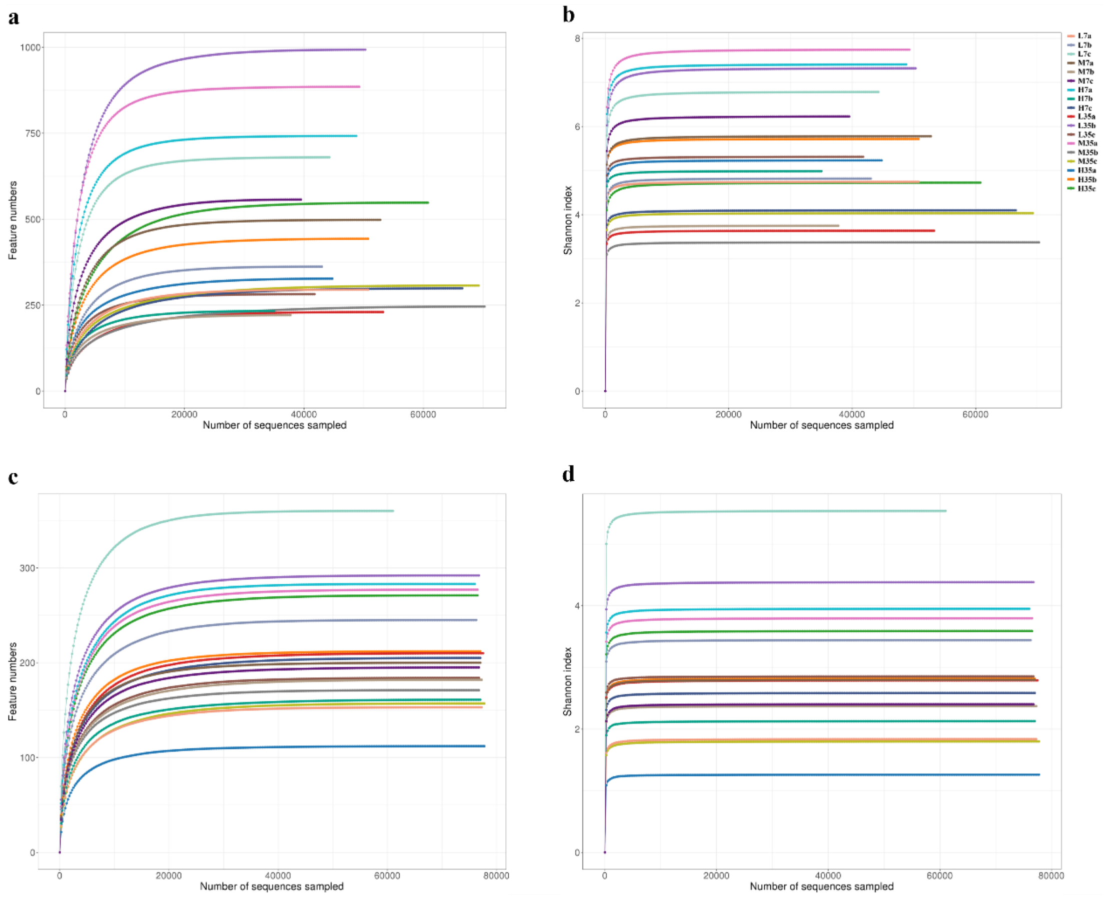

**Supplementary Table 1** The α-diversity of the bacterial and fungal community during fermentation of BBP-meju with different salt concentrations.

| Samples | OTUs | | ACE | | Chao1 | | Simpson | | Shannon | | Coverage | |
|---|---|---|---|---|---|---|---|---|---|---|---|---|
| | Bacteria | Fungi | Bacteria | Fungi | Bacteria | Fungi | Bacteria | Fungi | Bacteria | Fungi | Bacteria | Fungi |
| L7a | 295 | 153 | 295.56 | 153.75 | 295.96 | 153.45 | 0.91 | 0.36 | 4.74 | 1.84 | 99.98% | 99.96% |
| L7b | 362 | 245 | 362.18 | 245.44 | 362.43 | 245.24 | 0.92 | 0.67 | 4.82 | 3.44 | 99.96% | 99.97% |
| L7c | 221 | 360 | 221.23 | 360.14 | 221.13 | 360.12 | 0.8 | 0.95 | 3.75 | 5.54 | 99.97% | 99.98% |
| M7a | 742 | 200 | 742.43 | 200.38 | 742.36 | 200.47 | 0.98 | 0.56 | 7.41 | 2.8 | 99.98% | 99.98% |
| M7b | 680 | 182 | 680.29 | 182.73 | 680.08 | 182.43 | 0.97 | 0.47 | 6.78 | 2.37 | 99.99% | 99.97% |
| M7c | 557 | 195 | 557.36 | 195.17 | 557.73 | 195.61 | 0.95 | 0.48 | 6.23 | 2.4 | 99.93% | 99.96% |
| H7a | 498 | 283 | 498.07 | 283.82 | 498.43 | 283.16 | 0.95 | 0.75 | 5.78 | 3.95 | 99.98% | 99.97% |
| H7b | 233 | 161 | 233.13 | 161.77 | 233.66 | 161.85 | 0.95 | 0.43 | 4.99 | 2.13 | 99.96% | 99.96% |
| H7c | 299 | 205 | 299.21 | 205.34 | 299 | 205.33 | 0.86 | 0.52 | 4.1 | 2.58 | 99.94% | 99.98% |
| L35a | 230 | 277 | 230.36 | 277.17 | 230.28 | 277.22 | 0.8 | 0.74 | 3.64 | 3.8 | 99.96% | 99.98% |
| L35b | 327 | 212 | 327.25 | 212.44 | 327.34 | 212.04 | 0.94 | 0.55 | 5.23 | 2.82 | 99.98% | 99.95% |
| L35c | 282 | 271 | 282.19 | 271.73 | 282.34 | 271.77 | 0.95 | 0.69 | 5.31 | 3.59 | 99.95% | 99.92% |
| M35a | 993 | 112 | 993.73 | 112.43 | 993.07 | 112.93 | 0.97 | 0.24 | 7.32 | 1.26 | 99.96% | 99.93% |

| | | | | | | | | | | | |
|---|---|---|---|---|---|---|---|---|---|---|---|
| M35b | 885 | 171 | 885.14 | 171.36 | 885.53 | 171.61 | 0.98 | 0.48 | 7.74 | 2.39 | 99.95% | 99.97% |
| M35c | 548 | 157 | 548.16 | 157.53 | 548.82 | 157.87 | 0.84 | 0.36 | 4.73 | 1.8 | 99.97% | 99.92% |
| H35a | 443 | 210 | 443.45 | 210.06 | 443.54 | 210.43 | 0.96 | 0.57 | 5.72 | 2.79 | 99.97% | 99.99% |
| H35b | 246 | 292 | 246.29 | 292.36 | 246.13 | 292.81 | 0.74 | 0.82 | 3.37 | 4.38 | 99.98% | 99.93% |
| H35c | 307 | 184 | 307.43 | 184.99 | 307.64 | 184.09 | 0.84 | 0.6 | 4.03 | 2.85 | 99.98% | 99.95% |

ACE: Abundance-based Coverage Estimator.
